# Development and Implementation of the National Cancer Institute’s Food Attitudes and Behaviors Survey to Assess Correlates of Fruit and Vegetable Intake in Adults

**DOI:** 10.1371/journal.pone.0115017

**Published:** 2015-02-23

**Authors:** Temitope O. Erinosho, Courtney A. Pinard, Linda C. Nebeling, Richard P. Moser, Abdul R. Shaikh, Ken Resnicow, April Y. Oh, Amy L. Yaroch

**Affiliations:** 1 University of North Carolina at Chapel Hill, Chapel Hill, North Carolina, United States of America; 2 Gretchen Swanson Center for Nutrition, Omaha, Nebraska, United States of America; 3 U.S. National Cancer Institute, Rockville, Maryland, United States of America; 4 University of Michigan, School of Public Health, Ann Arbor, Michigan, United States of America; 5 Clinical Monitoring Research Program, SAIC-Frederick, Inc., National Cancer Institute-Frederick, Frederick, Maryland, United States of America; University of Brescia, ITALY

## Abstract

**Background:**

Low fruit and vegetable (FV) intake is a leading risk factor for chronic disease globally as well as in the United States. Much of the population does not consume the recommended servings of FV daily. This paper describes the development of psychosocial measures of FV intake for inclusion in the U.S. National Cancer Institute’s 2007 Food Attitudes and Behaviors Survey.

**Methods:**

This was a cross-sectional study among 3,397 adults from the United States.

Scales included conventional constructs shown to be correlated with fruit and vegetable intake (FVI) in prior studies (e.g., self-efficacy, social support), and novel constructs that have been measured in few- to- no studies (e.g., views on vegetarianism, neophobia). FVI was assessed with an eight-item screener. Exploratory factor analysis, Cronbach’s alpha, and regression analyses were conducted.

**Results:**

Psychosocial scales with Cronbach’s alpha ≥0.68 were self-efficacy, social support, perceived barriers and benefits of eating FVs, views on vegetarianism, autonomous and controlled motivation, and preference for FVs. Conventional scales that were associated (p<0.05) with FVI were self-efficacy, social support, and perceived barriers to eating FVs. Novel scales that were associated (p<0.05) with FVI were autonomous motivation, and preference for vegetables. Other single items that were associated (p<0.05) with FVI included knowledge of FV recommendations, FVI “while growing up”, and daily water consumption.

**Conclusion:**

These findings may inform future behavioral interventions as well as further exploration of other potential factors to promote and support FVI.

## Introduction

Diets high in fruits and vegetables (FVs) are associated with reduced risk for obesity and several chronic diseases [1–5]. National guidelines recommend that adults in the United States (U.S.) consume 7–13 servings (3½-6½ cups) of FVs daily, depending on sex and activity level [5,6]. However, surveillance studies report that most U.S. adults consume less than recommended amounts [7–9]. Understanding correlates of fruit and vegetable intake (FVI) are critical for developing and testing effective FVI interventions.

Studies report that psychosocial factors are associated with adults’ FVI [10–16]. For instance, greater perceived access and availability of FVs are associated with higher FVI [11,17,18]. Having positive attitudes toward FVs, believing in their health importance, liking their taste, having greater self-efficacy to eat them, and being knowledgeable of the FV recommendations are also associated with higher FVI in adults [10,12–14,16]. Measures for assessing psychosocial constructs related to FVI vary, and many assess a limited number of psychosocial constructs [19–23]. Therefore, the items developed for the current survey drew from multiple traditional behavioral theories such as the Social Cognitive Theory, Self-Determination Theory, Theory of Reasoned Action, and Health Belief Model, as well as novel items that are not specified in established behavioral theories [24–28]. Furthermore, although some measures have been developed for use with specific population subgroups such as low-income adults [13,29], but few measures for assessing psychosocial constructs have been specifically developed and tested in a national sample of U.S. adults [22].

This paper describes the development of psychosocial measures for inclusion in the U.S. National Cancer Institute’s (NCI) Food Attitudes and Behaviors (FAB) Survey. The survey included assessment of both conventional constructs that have been shown to be correlated with FVI in prior studies (e.g., barriers, self-efficacy, social support), and novel constructs that have been measured in few- to- no studies that specifically assess attitudes and behaviors in relation to food (e.g., views on vegetarianism, neophobia). First, the paper describes the process of identifying, selecting, and testing the psychometric properties of the survey items. Second, an evaluation of the psychosocial constructs’, including scales and single items, and the association with FVI will be reported.

## Methods

### Design

**Development and Pilot-testing of the FAB Survey**. Survey items were selected based on an extensive literature review [15] in which we identified conventional psychosocial constructs from cross-sectional and longitudinal studies that were significantly associated FVI. Most constructs and associated survey items (both existing and new items) included on the survey were based on common health behavior theories as stated above. Some novel scales and items that were not related to existing theories and/or had not been examined specifically with regard to FV attitudes and behaviors were included in the survey. A content validity review was conducted by nutrition, public health, and health behavior experts. Extensive cognitive interviewing was conducted with 68 adults to assess comprehension of survey items, and adjustments were made iteratively to ensure the items were understandable.

Psychometric testing of the survey was conducted in a pilot study (N = 579 adults). Based on pilot findings, some survey items were retained for use in scales and as single items, others were dropped, and some items were modified for use in the larger main implementation study (described below). Findings regarding reliability estimates from the pilot were consistent with the larger main implementation study, thus we do not report details from the pilot here.

### The Main FAB Implementation Study and Sample

The final FAB Survey [30] was comprised of 65 items that assessed food attitudes, beliefs and preferences, social support, knowledge, perceived access to FVs, food shopping behaviors, physical activity, perceived health, demographic characteristics, and FVI [31–34]. The study was approved by the National Cancer Institute’s institutional review board and passed through clearance at the Office of Management and Budget (OMB). The survey was administered to adults ages ≥18 years across the U.S. September-December, 2007. Respondents were selected from the Synovate Consumer Opinion Panel (http://www.ipsos.com/) using stratified random sampling, with an oversampling of African Americans. The FAB Survey was mailed to 5,803 adults; 3,418 surveys were returned, yielding a response rate of 57%; 21 incomplete surveys were excluded, for a final sample of 3,397. Respondents received a thank you letter and $5 for completing the survey.

### Measures

The following describe the constructs and associated survey items. A full copy of the FAB Survey and associated materials can be found at http://cancercontrol.cancer.gov/brp/fab/.

**Psychosocial Constructs and Single Items on the FAB Survey**. Conventional psychosocial constructs included self-efficacy, social support, perceived barriers and benefits of eating FVs, and FV purchasing behaviors (Table 1). These constructs have been shown in prior studies to be strong correlates of FVI [15]. Self-efficacy (7 items) measured confidence to consume FVs. Social support (5 items) asked about family/friends support and encouragement in eating FVs. Perceived benefits (7 items) asked about perceptions of health benefits of FVs. Perceived barriers (14 items) included: access, high cost, and short shelf-life of FVs.

**Table 1:** Internal consistency for conventional constructs related to fruit and vegetable intake.

FV Constructs	Items for measuring construct	Items Kept	Items Excluded	# of items included	Cronbach’s Alpha
**Self-efficacy**	Confidence to:				
	Eat a healthy snack, like a fruit/ vegetable, when hungry	X		7	0.92
	Eat healthy foods, like fruit/vegetables, when tired	X			
	Eat healthy foods, like fruit/vegetables, when junk foods are in your house	X			
	Eat fruit instead of cake, cookies, candy, ice cream, or other sweets for dessert	X			
	Eat fruits/vegetables when family and friends are eating junk foods	X			
	Buy or bring FVs to eat at work	X			
	Snack on FVs rather than on junk foods while watching TV	X			
**Social Support**	My family/friends:			3	0.68
	Encourage me to eat FVs				
	Remind me not to eat junk food	X			
	Would say something if they saw I was not eating FVs	X			
	Often eat FVs when we are together		X		
	Would be willing to eat a vegetarian/vegetable-based meal		X		
**Perceived benefits**	If you eat plenty of FVs every day, how likely are you to:				
	Have more energy	X		7	0.91
	Live a long life	X			
	Control your weight	X			
	Look better (appearance)	X			
	Be regular (cleanse the body)	X			
	Feel good about yourself	X			
	I eat enough FVs to keep me healthy	X			
**Perceived barriers**	I don’t eat FVs as much as I would like because:				
	They cost too much	X		14	0.85
	They often spoil before I get a chance to eat them	X			
	They take too much time to prepare	X			
	They are not filling enough	X			
	I have trouble digesting them	X			
	My family doesn’t like them	X			
	I don’t know how to choose fresh FVs	X			
**Perceived barriers**	I don’t think of FVs when I’m looking for something to eat	X			
	They are too messy	X			
	I often forget to eat FVs because they are stored out of sight	X			
	Restaurants I go to don’t serve fruit	X			
	Restaurants I go to don’t serve vegetables	X			
	It is not easy for me to purchase FVs in my neighborhood	X			
	It is hard for me to eat more FVs because I don’t know how to prepare them	X			
	When I eat out, it is easy for me to get FVs		X		

**Abbreviations**: FVs denotes fruits and vegetables.

Novel psychosocial constructs included views on vegetarianism (6 items), autonomous (11 items) and controlled motivation (7 items), preference for FVs (36 items), and food neophobia (3 items) (Table 2). The development of items was exploratory and based on emerging evidence or in the case of motivation, had previously been explored with other behaviors (e.g., smoking). Autonomous motivation was defined as motivations for performing behaviors for which the rewards were internal to the individual, while controlled motivation were those that were based on the receipt of external rewards or punishment [25,26,35]. Food neophobia asked about reluctance to try new foods.

**Table 2:** Internal consistency for novel constructs related to fruit and vegetable intake.

FV Construct	Items for measuring construct	Items Kept	Items Excluded	# of Items included	Cronbach’s Alpha
**Views on Vegetarianism**	Dinner doesn’t seem right without meat as a main course	X		5	0.76
	After I eat a meal without meat, I still feel hungry	X			
	Vegetarians are a bit “different”	X			
	I think meals should include some meat	X			
	I just don’t understand how someone could be a vegetarian	X			
	My family/friends would be willing to eat a vegetarian/ vegetable-based meal		X		
**Autonomous motivation**	What motivates you to eat FVs:				
	To feel in control of my health	X		11	0.95
	I have a strong value for eating healthy	X			
	I personally believe it is a good thing for my health	X			
	I have carefully thought about it and believe it is very important for me	X			
	I would feel better about myself if I did eat a healthy diet	X			
	I would like to improve my physical health	X			
	An important choice I really want to make	X			
	Consistent with my life goals	X			
	Important for being as healthy as possible	X			
	To take responsibility for my own health	X			
	Important to treat my body with respect	X			
**Controlled Motivation**	What motivates you to eat FVs:				
	Others would be upset with me if I did not	X		7	0.89
	I feel pressure from others to eat FVs	X			
	I want others to approve of me	X			
	It’s easier to do what I am told than to think about it	X			
	I want others to see I can do it	X			
	I want to set a good example for my community	X			
	I don’t want to let others down	X			
	I want to set a good example for my family		X		
**Preference for FVs**	**Preference (like/dislike) for:**				
	a) apples, applesauce; b) bananas; c) pears; d) watermelon; e) other melon;	X		36	0.92
	f) peaches, nectarine, apricots; g) plums; h) grapes; i) oranges, tangerines;	X			
	j) strawberries; k) other berries; l) grapefruit; m) kiwi; n) cherries; o) mango, papaya; p) pineapple; q) dried fruit	X			
	**Preference (like/dislike) for:**				
	a) tomatoes, tomato sauce; b) broccoli; c) spinach (cooked);	X			
	d) collards, turnip greens, or mustard greens (cooked); e) string beans, green beans	X			
	f) asparagus; g) green, red, or yellow pepper; h) celery; i) cucumber; j) peas	X			
	k) lima, red, pinto, kidney, lentils, and other beans; l) squash, zucchini	X			
	m) Brussels sprouts; n) cauliflower; o) okra; p) corn; q) carrots; r) green salad	X			
	s) yams, sweet potatoes	X			
	t) baked potatoes, mashed potatoes, or potato salad		X		
**Neophobia**	I enjoy trying new foods	X		3	0.57
	When it comes to food, I’m a creature of habit. I eat the same things all the time	X			
	I am usually the first of my friends to try new food/nutrition products	X			

**Abbreviations**: FVs denotes fruits and vegetables.

Single items on the survey were either other behaviors or items that did not fit within a scale (i.e., low alphas) and included: physical activity (participation/non-participation for ≥30 minutes daily); smoking (never/former/current smoker); awareness and knowledge of FV recommendation; and two out of three items from the original food neophobia scale (see Table 2). Additional single items asked about “worry” (how much has worrying about your health led you to change the way you ate in the past year), and seasonality (do you tend to eat the same types of FVs all year round or tend to eat different types of FVs depending on the season?). Finally, respondents were asked about the amount (cups) of water they consumed daily, and how often they ate FVs while growing up.

**Fruit and Vegetable Intake**. The main outcome variable on the survey was FVI during the past month. This was assessed with an eight-item FV screener that was modified from the NCI FV screener [36], and validated using multiple 24-hour dietary recalls (adjusted correlation coefficients ranged from 0.39–0.57 for fruit, vegetable, and FV combined) [37]. Responses included ten frequency categories ranging from never to ≥5 times/day, and four portion size categories ranging from about ¼ cup to more than 2 cups. Responses were converted into servings, as defined by the MyPyramid 1992 dietary guidelines [36]. Total FVI was calculated as the sum of all items on the screener, excluding fried potatoes.

**Demographic Characteristics**. Demographic characteristics that were assessed included sex, age, race/ethnicity, highest level of education completed, income, and geographic region of residence.

### Analysis

Exploratory factor analysis was conducted using Mplus statistical software (v.5). Factor loadings helped inform the factor structures and determined items to retain within each scale. Items with factor loading lower than 0.3 were considered unsatisfactory items and were excluded from the scales, while items with factor loadings of ≥0.3 were typically kept in the scales [38].

Following exploratory factor analysis, internal consistency was assessed with the items that were retained after the factor analysis. Within each scale, an overall Cronbach’s alpha (α) was computed, and for each item, an index “α if item deleted” was computed. Scales with Cronbach’s α ≥0.68 were entered into regression models for further analysis.

Hierarchical linear regression was conducted using SAS (v.9.1, SAS Institute, Cary, NC) to evaluate associations between the psychosocial scales and single items with FVI. Five regression models were tested in a stepwise manner with statistical significance set at p<0.05 (two-sided): (1) sociodemographic variables, (2) lifestyle variables (physical activity, smoking status), (3) conventional scales (self-efficacy, social support, perceived barriers, perceived benefits), (4) novel scales (views on vegetarianism, autonomous and controlled motivation, preference/liking for FVs), (5) unscaled single items. All regression models incorporated sample weights to obtain population-level estimates. These weights were based on post-stratified U.S. Census values for sex, race/ethnicity, age, education, and income. Tests for collinearity were conducted for final regression models and no collinearity issues were found. Missing data, generally around 1% for all items, were imputed using the cyclic n-partition hot decks and predictive means matching method [39,40].

## Results

Sociodemographic characteristics of respondents are described in Table 3. Fifty-three percent were female, 27% non-Hispanic black, and 36% were 35–54 years old. Sixty percent had completed high school, 38% resided in the West, and 66% were overweight/obese.

**Table 3:** Sociodemographic characteristics of main implementation (unweighted frequencies and weighted percentages).

	n (%)
Sex	
Male	1300(47)
Female	2009(53)
Age (years)	
18–34	949(31)
35–54	1312(36)
≥ 55	1053(33)
Race/ethnicity	
Non-Hispanic white/Other	2368(73)
Non-Hispanic black	896(27)
Highest level of education completed	
< high school	408(14)
High school degree	2001(60)
College degree or higher	901(26)
Region	
Northeast	603(19)
Midwest	677(23)
West	1248(38)
South	493(20)
Body mass index	
≤ 24.9 kg/m^2^ (under/normal weight)	1031(32)
25.0–29.9 kg/m^2^ (overweight)	1094(34)
≥ 30.0 kg/m^2^ (obese)	1102(33)

Scales with Cronbach’s α≥0.68 were self-efficacy, social support, perceived barriers and benefits of eating FVs, views on vegetarianism, autonomous and controlled motivation, and preference for FVs (Tables 1 and 2). Food neophobia had a Cronbach’s α<0.68, thus the scale was excluded from the regression models while the strongest single items were included.

Table 4 describes associations between the psychosocial scale and single items and FVI. The final model that included only psychosocial scales and single items that were significant in Model Five explained 31% of the variance in FVI. Lower FVI (p<0.01) was reported by respondents who reported not participating in physical activity (β = 0.16), or perceived barriers that prevented them from eating FVs (β = -0.14). Lower FVI (p<0.05) was also reported by respondents who said they did not eat fruits (β = -0.07) or vegetables (β = -0.08) while growing up, were a “creature of habit” (i.e., eating the same foods all the time) (β = -0.03), and did not know daily FV recommendations (β = -0.12).

**Table 4:** Associations of conventional and novel psychosocial constructs and single items with fruit and vegetable intake (excluding fried potatoes)^a^.

	**Model 1**	**Model 2**	**Model 3**	**Model 4**	**Model 5**	**Final Model**
	**β (p-value)**	**β (p-value)**	**β (p-value)**	**β (p-value)**	**β (p-value)**	**β (p-value)**
**Sociodemographic variables**						
Sex (female vs. male)	0.13(<0.001)	0.13(<0.001)	0.04(0.29)	-0.03(0.46)	-0.14(0.21)	
Age	0.09(<0.001)	0.11<0.001)	0.06(0.02)	-0.02(0.55)	-0.05(0.57)	
Race/ethnicity						
Hispanic	0.29(0.03)	0.28(0.02)	0.14(0.21)	0.11(0.29)	0.10(0.32)	
NH Black	0.20(<0.001)	0.21(<0.001)	0.01(0.90)	-0.01(0.86)	0.03(0.59)	
NH Other	0.34(<0.001)	0.32(<0.001)	0.20(0.03)	0.15(0.10)	0.12(0.17)	
NH White	Reference	Reference	Reference	Reference	Reference	
Education	0.10(<0.001)	0.07(0.00)	0.05(0.02)	0.04(0.09)	0.01 (0.55)	
Income	0.00(0.94)	-0.00(0.50)	-0.01(0.20)	-0.00(0.27)	-0.00 (0.20)	
Region						
Midwest	-0.00(0.96)	-0.01(0.85)	0.01(0.83)	0.03(0.65)	0.07(0.24)	
Northeast	0.04(0.50)	0.05(0.47)	0.06(0.36)	0.08(0.15)	0.12(0.03)	
South	-0.07(0.30)	-0.05(0.42)	-0.06(0.27)	-0.06(0.28)	-0.02(0.77)	
West	Reference	Reference	Reference	Reference	Reference	
**Lifestyle variables**						
Physical activity *(participation vs. non-participation)*		-0.48(<0.00)	-0.30(<0.001)	-0.26(<0.001)	-0.16(<0.001)	-0.16(<0.001)
Smoking status *(current vs. former and never smoker)*		-0.02(0.41)	-0.01(0.74)	0.01(0.80)	0.00(0.93)	----
**Conventional Psychosocial Constructs**						
Self-efficacy			0.30(<0.001)	0.20(<0.001)	0.14(<0.001)	0.15(<0.001)
Social Support			0.13(<0.001)	0.11(<0.001)	0.08(<0.001)	0.08(<0.001)
Barriers			-0.31(<0.001)	-0.23(<0.001)	-0.13(<0.001)	-0.14(<0.001)
Benefits			0.04(0.08)	-0.07(0.02)	-0.03(0.21)	----
**Novel Psychosocial Constructs**						
Vegetarianism views				0.03(0.19)	-0.02(0.44)	----
Autonomous motivation				0.17(<0.001)	0.08(<0.001)	0.06(0.01)
Controlled motivation				-0.02(0.35)	-0.03(0.23)	----
Preference for fruit				0.44(<0.001)	0.20(0.06)	----
Preference for vegetable				0.55(<0.001)	0.31(0.01)	0.37(<0.001)
**Singe Items**						
Creature of habit (eating same things all the time)					-0.04(0.03)	-0.03 (0.04)
Unwillingness to try new foods					-0.04(0.06)	----
Not attentive to government FV recommendations					-0.01(0.46)	----
Worry about one’s health					0.01(0.55)	----
Drink several cups of water daily					0.14(<0.001)	0.13(<0.001)
Seasonality *(varying FV intake by season vs. eating same types year round)*					0.18(<0.001)	0.19(<0.001)
Did not eat fruit when growing up *(negative responses)*					-0.07(<0.001)	-0.08(<0.001)
Did not eat vegetable when growing up *(negative responses)*					-0.08(<0.001)	-0.08(<0.001)
No knowledge of FV recommendations *(wrong responses vs. correct)*					-0.12(<0.001)	-0.12(<0.001)
Eat more FV than other people I know					0.16(<0.001)	0.16(<0.001)
Often encourage my family/friends to eat FV					0.02(0.33)	----
R^2^	0.02	0.06	0.21	0.25	0.32	0.31
R^2^ change	---	0.04	0.15			

Abbreviations: FVs denotes “fruits and vegetables”; NH denotes “Non-Hispanic”.

^a^Scales that demonstrated good internal consistency (Tables 1 and 2) with Cronbach’s alpha ≥.68 were included in the regression analyses Single items that did not fit within a scale were also entered into the regression models.

Higher FVI (p<0.05) was reported by respondents reporting greater self-efficacy (β = 0.15), social support (β = 0.08), and autonomous motivation for consuming FVs (β = 0.06), as well as a preference for vegetables (β = 0.37). Higher FVI (p<0.01) was also reported by respondents that consumed different FVs seasonally (β = 0.19), perceived that they ate more FVs than other people they knew (β = 0.16), and drank several cups of water daily (β = 0.13).

## Discussion

The FAB Survey measured conventional psychosocial constructs related to FVI (i.e., self-efficacy, social support, perceived barriers and benefits of eating FVs) that have been shown to be strong correlates of FVI [13,15,19,20,41,42]. Additionally, the survey included the development and assessment of novel psychosocial constructs related to FVI (i.e., vegetarianism, autonomous and controlled motivation, food neophobia, and preference for FVs). All scales, except food neophobia, demonstrated good internal consistency with Cronbach’s α ≥0.68.

With regard to outcomes, adults consumed more FVs if they reported having greater self-efficacy and social support but consumed fewer FVs if they perceived more barriers. Other studies have reported similar findings [13,41–43].

Consistent with other studies [14,44], this study demonstrated that adults having preferences for a greater selection of vegetables ate more FVs. In addition, adults with higher autonomous motivation for consuming FVs also ate more FVs. According to the Self-Determination Theory, enhancing autonomous motivation is likely to result in sustainable behaviors (in this case, greater FVI), because it is self-driven, and not influenced by external pressures such as rewards/punishment [25,26,43]. Nevertheless, the effects of autonomous motivation on FVI have varied across studies [35,45]. Assessing and intervening on autonomous motivation in relation to FVI is relatively new, hence we termed it as novel; prior studies have focused mostly on autonomous motivation for other behaviors (i.e., smoking cessation) [46–48].

With regard to single items, the current study showed that FVI was greater among adults that reported eating more FVs than other people they knew, seasonality effects, and greater water consumption. However, lower FVI was reported by adults who did not eat FVs frequently while growing up, and those not knowledgeable of the daily recommendation. The current study highlights the need to introduce individuals to FVs early in life, given that these behaviors tend to track into adulthood, where they play a significant role in health and well-being [49–51]. In addition, this study helps provide foundational information in elucidating the role psychosocial constructs may play and their potential associations with FVI and how they can be harnessed and applied in behavioral interventions. Specifically, results from this study underscore the need to continue examining and intervening on “usual suspect” FV constructs (e.g., self-efficacy and social support) but that we should also continue to test novel constructs; namely ones explored in this analysis (e.g., seasonality, water intake, fruit and vegetable behaviors when growing up), as well as others, either derived from existing theories or as they are discovered through conducting research. Next steps include application of this information in intervention research.

This study has some limitations. The cross-sectional design does not allow for assessment of causality between the psychosocial scales, single items, and FVI. Data were collected via self-report from participants and subject to recall bias. Due to budget constraints and the declining response rates to random-digit-dial telephone surveys [52], the samples for both the pilot and implementation studies were drawn from a consumer opinion panel. This approach has been used successfully with other health survey, such as the Styles [53]. Nevertheless, the FAB sample was weighted based on post-stratified 2000 U.S. Census values for sex, race/ethnicity, age, education, and income. Specifically for this sample, eligible participants were selected to be representative of the U.S. population and previous research that has compared panel and random digit dial results have shown comparability, indicating that panel studies are a viable alternative for data collection, especially as telephone random digit dial response rates are dropping [54]. Lastly, some consider the theory behind hot deck imputation underdeveloped [55], however, we used cyclical n-partition hot deck imputation, a method that retains the semiparametric features of the data and have no strong assumption required about distribution shapes [56,57]. Strengths of the study are the large sample size and oversampling of African Americans. The FV screener was tested for reliability and validated using 24-hour dietary recalls [37].

## Conclusions

Most U.S. adults continue to not meet FV recommendations. Measures for assessing psychosocial constructs related to FVI vary and many assess a limited number of constructs. Few existing measurement tools have been tested among a national sample of U.S. adults. This paper describes the development and testing of FV-related measures among a sample of U.S. adults. It describes both conventional and novel correlates of FVI, which augments the literature in this area. Items and scales from the FAB Survey can be utilized and/or adapted by researchers interested in measuring FVI. It can also help inform behavioral interventions.
